# Depression among People Living With HIV/AIDS Undergoing Antiretroviral Therapy at a Tertiary Care Centre: A Descriptive Cross-sectional Study

**DOI:** 10.31729/jnma.8157

**Published:** 2023-05-31

**Authors:** Sunil Kumar Shah, Richa Sinha, Srijana Bhurtel, Gobinda Kandel

**Affiliations:** 1 Department of Psychiatry, Bharatpur Hospital, Bharatpur, Chitwan, Nepal; 2 Department of Internal Medicine, Bharatpur Hospital, Bharatpur, Chitwan, Nepal

**Keywords:** *depression*, *HIV*, *prevalence*

## Abstract

**Introduction:**

HIV affects mental health in multiple ways, including its direct pathophysiological effects, associated stigma, impacts on social, and economic dimensions, medications used for long durations and multiple secondary physical health issues that affect the clients and comorbid substance use. In the post-COVID era, in our socio-cultural and geographical context, depression among these populations needs assessment to evaluate their mental health care needs. The objective of this study was to find out the prevalence of depression among people living with HIV/AIDS undergoing antiretroviral therapy at a tertiary care centre.

**Methods:**

This was a descriptive cross-sectional study conducted at a tertiary care centre from December 2021 to November 2022 after taking ethical approval from Institutional Review Committee (Reference number: 078/79-006) from the same institute. Convenience sampling method was used. Clients 18 years and above under antiretroviral therapy were included and those acutely medically ill were excluded. The patient health questionnaire (PHQ-9) was used as a self-administered, valid, screening tool for the assessment of depressive symptoms. Point estimate and 95% Confidence Interval were calculated.

**Results:**

Among 183 participants, the prevalence of depression was 19 (10.4%) (5.98-14.82, 95% Confidence Interval).

**Conclusions:**

Depression was found higher among people living with HIV/AIDS as compared to the other studies done in similar settings. Assessment and timely management of depression could be an important step in improving lives and the effectiveness of HIV/AIDS intervention efforts, ultimately improving access to mental health care and universal health coverage.

## INTRODUCTION

Depression is a major contributor to the global burden of disease and the treatment gap is huge in low and middle-income countries.^1^ HIV/AIDS being universally better-taken care of, mental health burden raises concern.^2,3^ Pathophysiological effects, stigma, socioeconomic burden, medicines and secondary medical issues are multiple ways HIV affects mental health. While mental illnesses and substance use increases the risk and complication of HIV and puts forward further challenges.^4^ Since the pandemic, the need to address mental health needs has been highlighted for wider coverage of the quality treatment of depression.

Depression warrants attention due to its high burden and significant impact.^5^

The objective of this study was to find out the prevalence of depression among people living with HIV/AIDS (PLHIV) undergoing antiretroviral therapy at a tertiary care centre.

## METHODS

This was a descriptive cross-sectional study conducted at a tertiary hospital antiretroviral therapy (ART) centre. This study was conducted from 1 December 2021 to 30 November 2022 after taking ethical approval from Institutional Review Committee (Reference number: 078/79-006) from Bharatpur Hospital, Bharatpur, Chitwan, Nepal. PLHIV patients of age 18 years and above who are under ART therapy were included in the study. Patients with acute medical health issues, bipolar and psychosis were excluded. Informed consent was taken. Personal interviews were taken and data was collected using proforma and PHQ-9. Convenience sampling method was used. The sample size was calculated by using the following formula:


$$\begin{array}{ll} \text{n} & ={\text{Z}}^{2}\times \frac{\text{p}\times \text{q}}{{\text{e}}^{\text{2}}}\\ & =1.96^{2}\times \frac{0.146\times 0.854}{0.06^{2}}\\ & =133 \end{array}$$

Where,

n = minimum required sample size
Z = 1.96 at 95% Confidence Interval (CI)
p = prevalence taken as 14.6% from previous study^6^
q = 1-p
e = Margin of error, 6%

The calculated sample size was 133. Adding 10% nonresponse rate, obtained sample size was 147. However, we took 183 sample.

Data collected with a self-structured questionnaire and PHQ-09 was checked for completeness and coded with serial numbers. The PHQ-9 was used as a self-administered, valid, screening tool for the assessment of the severity of depressive symptoms. PHQ-9 includes 9 items which focus on the Diagnostic and Statistical Manual of Mental Disorders, 4^th^ edition (DSM-IV) for major depressive disorder. The questionnaire assesses how often the participants had been disturbed by any of the 9 items during the last 2 weeks. Each item of PHQ-9 was scored on a scale of 0-3 (0 =not at all; 1 = several days; 2 = more than a week; 3 = nearly every day). The PHQ-9 total score ranges from 0 to 27 (scores of 5-9 are classified as mild depression; 10-14 as moderate depression; 15-19 as moderately severe depression; >20 as severe depression. The test discriminates well between people with and without Major depressive disorder (MDD).^7-9^ The PHQ-9 was found to have acceptable diagnostic properties for detecting major depressive disorder for cut-off scores between 8 and 11.^10^ The PHQ-9 with a cut-off score of 10 or above to identify major depression can be used regardless of age.^11^ Data were entered and analysed using IBM SPSS version 21.0. Point estimate and 95% CI were calculated.

## RESULTS

Among 183 participants, the prevalence of depression was 19 (10.4%) (5.98-14.82, 95% CI). The mean age was 41.37±14.54 years. The majority of the clients, 8 (42.1%) with depression were from the age category 30-40. More females were found depressed 11 (57.9%) (Table 1).

**Table 1:** Demographic characteristics of clients with depression (n= 19).

Variables	
**Age (Years)**	**n (%)**
<30	4 (21.10)
30-40	8 (42.10)
40-50	3 (15.78)
50-60	2 (10.52)
>60	2 (10.52)
**Gender**	
Female	11 (57.89)
Male	8 (42.10)
**Marital status**	
Married	17 (89.47)
Never married	2 (10.52)
**Religion**	
Hindu	18 (94.73)
Muslim	1 (5.26)
**Ethnicity**	
Brahmin	3 (15.8)
Chhetri	3 (15.8)
Madhesi	2 (10.52)
Gurung	1 (5.26)
Magar	1 (5.26)
Newar	1 (5.26)
Others	6(31.57)
**Occupation of the head of the family**	
The clerical, shop owner, farmer	14 (73.68)
Semi-skilled worker	2 (10.52)
Unemployment	2 (10.52)
Unskilled worker	1 (5.26)
**Income of the family (NRs)**	
14,551 to 24,350	4 (21.05)
24,351 to 36,550	4 (21.05)
4851 to 14,550	9 (47.36)
97,451 or more	1 (5.26)
Less than 4850	1 (5.26)
**Economic status**	
Lower (V)	1 (5.26)
Upper lower (iv)	12 (63.15)
Lower middle (iii)	6(31.57)
**Education**	
Middle school	4 (21.05)
Primary school or literate	5 (26.31)

Of more clients on the TLD regimen, 15 (78.9%) were found to have depression. Of those who were identified with HIV for more than 10 years, 9 (47.4%) were more among the sufferers. Those with family members also having HIV suffered more, 12 (63.2%). Clients reporting facing discrimination counted more among depressed ones, 10 (52.6%) (Table 2).

**Table 2:** Clinical and social factors of clients (n= 19).

Variables	n (%)
**ART regimen**	
^*^TLD	15 (78.94)
^†^TLE	4 (21.05)
**HIV identified years (in years)**	
1-5	6 (31.57)
5-10	4 (21.05)
>10	9 (47.36)
**Initiation of ART in duration (in years)**	
1-5	7 (36.84)
5-10	4 (21.05)
>10	8 (42.10)
**Distance to ART centre from home (in Km)**	
0-10	1 (5.26)
10-20	6 (31.57)
20-30	4 (21.05)
30-40	3 (15.78)
40-50	1 (5.26)
>50	4 (21.05)
**HIV-related complications in the last year**	
URTI	2 (10.52)
**Family history of HIV**	12 (63.15)
**Been discriminated against due to your HIV status**	10 (52.63)

* TLD= Tenofovir disoproxil Lamivudine, Dolutegravir

† TLE= Tenofovir disoproxil, Lamivudine, Efavirenz

## DISCUSSION

In our study, depression was prevalent among 19 (10.4%) of 183 (5.97-14.82, 95 % CI) HIV patients under ART. As per the national mental health survey of Nepal, lifetime prevalence and current prevalence of depressive disorders among the adult population were found 2.9 (2.3-3.7, 95% CI) and 1.0 (0.8-1.4 at, 95% CI) respectively.^12^ Prevalence of depression among the study participants is clearly several folds higher than among the general population of Nepal. In 2020, communities affected by COVID-19 faced mental illnesses like major depression and anxiety.^13^ Similar findings have been reported across several studies. The prevalence of depression among adult HIV/AIDS patients on ART was 14.6% (10.9-18.2, 95% CI) in a study of 2015 in Ethiopia. In this study, a positive depression screen was defined as a PHQ-9 score greater than 9.^6^ Likewise, in a comparative cross-sectional study conducted in Ethiopia in 2018, the prevalence was significantly higher in people with HIV/AIDS compared with the community sample (16.6% vs 12.3%).^14^

Some other studies had shown still a higher prevalence of depression among PLHIV. The prevalence of depression was 26.7% (20.6-33.7, 95% CI) in a study done from 2014 to 2015 in Cameroon.^15^ In another study in Nepal done in 2014, 29.6% of the participants had depressive symptoms (CESD).^16^ Similarly, in a hospital-based study in Karnataka in 2018, 108 (33.5%) PLHIV had depression according to PHQ 9 questionnaire.^17^

While some other studies report a much higher prevalence of depression among PLHIV. In a study done in Nepal in 2019, depression was very prominent among HIV-positive patients, 40% of participants were found to have depression.^18^ While the report from the pilot study of the national mental health survery states that, among adults aged 18 years and above, major depressive disorder (current) was found among 3.4%.^1^ In addition, a study in a medical college in Delhi, published in 2014 found that the prevalence of depression in patients with HIV under ART was 58.75%.^19^ Likewise in a study done in Indonesia in 2018, depressive symptoms were exhibited by 50.9% of participants.^20^

Wide variation in the prevalence of depression among PLHIV under ART has been observed across studies in various parts of the world. A multitude of factors could be contributing to the variations in the prevalence of depression among participants. Tools cut-off scores used for assessment of depression could be one factor contributing to the differences in prevalences. The impact of HIV on multiple dimensions of the affected, like social, economic, psychological, physical etc could be the reason behind this finding. Lately, the pandemic has affected lives significantly causing a rise in various social, economic, physical and mental health issues.^21,22^ Mental health plays a critical role in the acquisition of HIV and PLWH experience higher rates of mental disorder.^23^

The limitation of the study was that it was conducted at a hospital. Convenient sampling technique was used.

## CONCLUSIONS

Depression was found highly prevalent among PLHIV under ART from the clinic as compared to the general population as reported by the National Mental health survey. So, regular assessment and strengthening the psychological support system might be suggestible.
